# *Mycobacterium avium* subsp. *Paratuberculosis* in Different Environmental Samples from a Dairy Goat Barn—Implications for Sampling Strategies for Paratuberculosis Diagnostic and Prevention

**DOI:** 10.3390/ani13101688

**Published:** 2023-05-18

**Authors:** Chris Pickrodt, Karsten Donat, Udo Moog, Heike Köhler

**Affiliations:** 1 Institute of Molecular Pathogenesis, Friedrich-Loeffler-Institut, Federal Research Institute for Animal Health, Naumburger Straße 96a, 07743 Jena, Germany; chris.pickrodt@fli.de; 2 Clinic for Obstretics, Gynaecology and Andrology for Large and Small Animal Science with Veterinary Ambulance, Justus-Liebig-University Gießen, Frankfurter Straße 106, 35392 Gießen, Germany; kdonat@thtsk.de; 3 Thuringian Animal Diseases Fund, Victor-Goerttler-Straße 4, 07745 Jena, Germany; umoog@thtsk.de

**Keywords:** small ruminants, Johne’s disease, MAP, environmental sampling, bedding, dust, feed, water, transmission, control program

## Abstract

**Simple Summary:**

Paratuberculosis is a chronic inflammatory disease leading to emaciation and production losses in ruminants. Important aspects of disease control are the detection of infected herds and environmental areas where contact between animals and the causative pathogen *Mycobacterium avium* subsp. *paratuberculosis* (MAP) is possible. Bedding, dust, feed, and water samples were collected from the barn of a paratuberculosis-infected dairy goat herd and analyzed for culturable MAP and MAP DNA. Cultivation was successful in 28 bedding and one dust sample, while MAP DNA was detected in 117 out of 256 samples from all materials. Samples collected from areas of high animal traffic, such as the milking parlor, were more likely to reveal positive results than adult and young goat areas. Positive culture results were also obtained from kidding pens, constituting this barn area as a possible infection site. Overall, environmental sampling may be suitable for the detection of MAP in goat herds and the identification of potential critical areas for pathogen transmission. These results should be taken into account for paratuberculosis control programs in goat herds to improve their efficiency and, thus, animal welfare.

**Abstract:**

Environmental samples are often used to classify the paratuberculosis status of cattle herds. The disease is caused by *Mycobacterium avium* subsp. *paratuberculosis* (MAP), predominantly through oral ingestion during infancy. In this explorative study, the presence of MAP was determined in the barn environment of a paratuberculosis-infected vaccinated dairy goat herd. A total of 256 bedding, dust, feed, and water samples were collected at eight time points and examined using culture and qPCR. Detection rates of both methods were compared, and factors determining MAP confirmation were identified. MAP was cultured from 28 bedding and one dust sample, while MAP DNA was detected in all materials (117/256). Samples from high animal traffic areas and those collected during the indoor season were more likely to yield positive culture and qPCR results. Cultivation of MAP from kidding pens indicated this area as a possible infection site. Dust proved to be the most suitable material for detecting MAP DNA, as bedding was for MAP culture. Environmental sampling was demonstrated to be an effective way to detect MAP in a dairy goat herd. qPCR results could confirm herd infection, while culture results provided insight into crucial areas for MAP transmission. These findings should be considered when designing farm-specific paratuberculosis control plans.

## 1. Introduction

Paratuberculosis is a chronic inflammatory intestinal disease of major economic importance in domestic ruminants. It is caused by an infection with *Mycobacterium avium* subsp. *paratuberculosis* (MAP) which usually occurs in the first days of life. The slow disease progression results in a long incubation period until symptoms such as diarrhea, decreasing milk yield, and emaciation up to death appear. Possible routes of transmission include oral ingestion of the pathogen, inhalation, or in-utero infection [1,2,3,4,5,6].

MAP is shed with the feces of infected animals and can remain infectious in the environment for almost one year, even outside a suitable host [1,7]. Detection of MAP in samples taken from the immediate environment of the animals can be exploited in two directions. First, when applied in the monitoring of paratuberculosis, this approach can be used for the identification of MAP-positive herds, ensuring a high probability of truly positive results. This is an important aspect of many control programs [8,9,10]. Environmental sampling for MAP detection, whether molecular biological or cultural, is considered to be cost-effective and highly specific [10,11,12]. Secondly, this diagnostic approach can identify areas with a high bacterial load that may serve as infection sources for susceptible animals in the herd. This can be valuable information in the framework of veterinary risk assessment within a herd aiming to improve hygiene and management.

Most studies concerning MAP detection in environmental samples have been conducted in cattle farms. Due to the partly considerable differences in the husbandry conditions of cattle and small ruminants, it is not possible to transfer the results obtained to other animal species without further verification. Only one previous study focused on grazing sheep flocks, including some combined herds of sheep and goats [13]. Systematic environmental sampling in dairy goat barns has not yet been conducted.

Different materials have been analyzed for the presence of MAP. As the pathogen is shed with feces, most studies conducted focused on manure samples. However, MAP has also been found in dust, water, feed, and soil samples from in and outside the barn [9,10,11,12,13,14,15,16,17]. A comparison between different matrices collected in the same barn to determine which is best suited for MAP detection in goat barns has been lacking. Barn areas with high animal traffic, such as alleyways and the milking parlor, revealed the highest incidence of MAP-positive samples in several studies in cattle, whereas calving pens and youngstock areas were less affected [9,10,11,12,18]. In this regard, both culture and qPCR appear to be appropriate detection methods and comparable in terms of their performance [14,15,16]. However, herds with a low prevalence may be misclassified as uninfected due to negative environmental samples caused by intermittent pathogen shedding or dilution as well as dissemination effects [17]. For this reason, repeated collection of multiple environmental samples is recommended to increase sensitivity [9,19,20].

The approach of drawing implications for disease management from environmental sampling results has been rarely investigated in cattle [21] but so far not in goats. The main objective during youngstock rearing should always be to prevent exposure of kids, lambs, and calves to MAP as long as possible. Although the minimal infectious dose has not yet been determined, the later exposure occurs, and the less MAP is taken up, the lower the likelihood for successful infection and or fast clinical expression may be [1,22,23]. High-risk areas for pathogen transmission should be identified, results critically evaluated, and measures for MAP reduction and contact minimization implemented in every individual farm-level paratuberculosis control plan.

Here, we present the results of an explorative study conducted on one dairy goat farm. It focused on MAP detection in environmental samples from a goat barn, as previously shown for cattle and sheep farms. The objective was to evaluate the suitability of different sample materials, sites, seasons, and analysis methods (qPCR and culture) for identifying a paratuberculosis-infected goat herd. In addition, the study aimed at determining crucial barn areas for exposure of kids to MAP to enable their integration into management strategies for effective disease control.

## 2. Materials and Methods

### 2.1. Animal Welfare and Legislation

The study was approved by the Animal Health and Welfare Unit of the Thuringian State Office for Consumer Protection (file reference: 2684-04-04-BFI-20-103) and carried out in accordance with European and national laws. Every effort was made to minimize suffering.

### 2.2. Study Herd

The study was conducted in an organic dairy goat farm in Thuringia, Germany, between 2020 and 2022. The herd was affected by paratuberculosis. MAP was first confirmed culturally in 2018 in different tissues of a clinically affected goat after diagnostic necropsy. Because of numerous clinical cases, all adult goats and the annual female youngstock were subsequently vaccinated with an inactivated vaccine (Gudair, CZ Vaccines, O Porriño, Spain), while goats showing severe clinical signs of paratuberculosis were culled.

Young and adult goats were kept in loose housing on deep litter in different open pens within the same building. The rotary milking parlor was located in a separate part of the building attached to the animal husbandry area. The parlor was entered from a passage from the waiting area adjacent to a part of the lactating goat pen. All lactating goats were driven into the waiting pen for milking twice a day.

Kids were raised at the farm for replacement. They were separated from the doe immediately after birth. About 20 age-grouped kids each were kept in temporary detached pens until reaching a body weight of about 15 kg. At about five months of age, the juvenile goats were moved from the group pen in the kid-rearing area to the juvenile goat pen on the opposite side of the building, and the kid pens were deconstructed afterwards. The kid-rearing area was cleaned and disinfected using white hydrated and slaked lime (calcium hydroxide) before new kid pens were built during the next kidding season.

Rotational grazing was performed during the grazing season between May and November. Young goats were turned out to graze after their first kidding. In 2020 and 2021, dried-off goats were grouped in separate pens approximately two months before kidding, whereas no dry goat/kidding pens were set up in 2022. The deep litter was removed from the pens before the end of each grazing season, followed by cleaning and disinfection using slake lime. In between, new bedding material was added every day as necessary to ensure clean udder skins and coats.

### 2.3. Study Design

**Figure 1:**
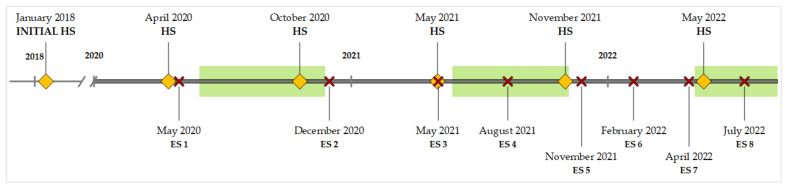
Sampling schedule for fecal and environmental samples between 2018 and 2022. HS = herd sampling (♦), ES = environmental sampling (✕). Grazing seasons are marked as green boxes.

Environmental samples were collected at eight environmental sampling events (ES) between 2020 and 2022. ES 1, 2, and 3 were conducted within a six-month interval, while the sampling frequency was enhanced to approximately every three months from ES 4 onwards (Figure 1).

Herd sampling to determine fecal shedding of MAP was first conducted in February 2018 and then biannually between 2020 and 2022 (Figure 1).

### 2.4. Environmental Sample Collection

The following nine sampling sites were defined: entrances to the barn, waiting area in front of the milking parlor, milking parlor, exit of the milking parlor, lactating goat pen (divided into four quadrants: Q1–Q4), dry goat/kidding pen(s), buck pen, juvenile goat pen, and goat kid pen(s). If more than one dry goat/kidding or goat kid pen existed at the same time, each pen was sampled individually. Four different materials (bedding, dust, water, feed) were collected from each site if available. Due to herd management, it was not possible to sample all locations or collect all different sampling materials at each ES. A detailed breakdown of all collected environmental samples (*n* = 256) at the respective ES and sampling sites is provided in Appendix A (Table A1).

Bedding material from approximately five spots within a whole pen or pen quadrant was combined into one composite sample. Deviating from this procedure, four individual samples were collected from kidding pens. Feed samples were collected out of racks or from the ground when forage was offered in front of the respective pen. Each feed and bedding sample was collected manually using a fresh glove. The samples contained between 50 and 250 g of forage or bedding with fecal material. The material was placed in a plastic bag, transferred to the laboratory, and stored at −20 °C. Dust samples were scraped from the surface of the barn facilities in a 50 mL tube using a scalpel. Water from drinking troughs was collected using a 1 L vial scooped through the trough. Dust and water samples were transferred to the laboratory and stored at room temperature until further processing.

### 2.5. Sample Preparation and Analysis

#### 2.5.1. Fecal Samples

Fecal samples from every goat older than one year were examined by bacterial culture according to the official manual of diagnostic procedures published by the Friedrich-Loeffler-Institute [24]. In short, 3 g of feces were decontaminated over 48 h at room temperature using 30 mL of 0.75% hexadecylpyridinium chloride monohydrate solution (Sigma Aldrich, Taufkirchen, Germany). The supernatants were discarded, and 200 µL of the remainder were transferred on each of three slopes of Herrold’s Egg Yolk Agar with Mycobactin J and Amphotericin, Nalidixic acid and Vancomycin (HEYM, Becton Dickinson, Sparks, MD, USA). Cultures were incubated at 37 °C for up to six months. Examination of bacterial growth was conducted every second week, starting after 42 days. The presence of MAP was confirmed by IS*900* PCR [25] of characteristic colonies.

#### 2.5.2. Bedding and Feed Samples

Samples were thawed overnight and processed according to Whittington et al. [7], modified as follows: 50 g of each sample was placed in a lockable plastic container. Then, 500 mL of sterile distilled water was added to cover the material completely. After intensively shaking by hand, containers were agitated in a shaking incubator overnight at 37 °C and 50 rpm. Stepwise, the liquid was poured off and collected in 50 mL vials, followed by centrifugation at 4200 rpm for 20 min. Two aliquots of 3 g each from the obtained pellet were portioned into two vials for further cultural and molecular biological examination. If the total pellet weight was below 6 g, the pellet was divided into two equal parts, and volumes of further additives were adjusted proportionally.

Based on the procedure applied to the fecal samples, the first aliquot of this pellet was processed for bacterial cultivation in the same way as described above using three slopes of HEYM (Becton Dickinson, Sparks, MD, USA). Examination of bacterial growth was conducted over six months, and the presence of MAP was confirmed by IS*900* PCR [25] of characteristic colonies. Based on the approach of Köhler et al. [26], a colony score (CS) from 0 to 5 was used to estimate MAP growth semi-quantitatively (0 = 0; 1 = 1–10; 2 = 11–20; 3 = 21–50; 4 = 51–100 individual colonies per slope; 5 = bacterial lawn). The week of appearance (WA) was also documented. For numerical estimation of the MAP concentration in the sample, a growth index (GI) for each slope was calculated using the formula:

GI = CS × 100/WA(1)

followed by determination of the mean of all inoculated slopes of the sample.

For qPCR analysis, 20 mL of sterile distilled water was added to the second aliquot of the pellet. After vigorous vortexing, the sample was left in an upright position for 20 min at room temperature for sedimentation. Then, 10 mL of the supernatant was centrifiltrated at 3000× *g* for 5 min using an ADIAFILTER (Adiagene, Bio-X Diagnostics S.A., Rochefort, Belgium) to concentrate bacteria and remove PCR-inhibiting substances. The resulting pellet was suspended in 500 µL sterile distilled water. Then, 300 mg zirconia/glass beads (Carl Roth GmbH + Co. KG, Karlsruhe, Germany) were added and the sample was placed in a Mixer Mill MM 400 (Retsch GmbH, Haan, Germany) for 10 min at 30 Hz to mechanically disrupt contained bacteria and centrifugated at 15,000× *g* for 5 min afterwards. DNA extraction was performed from the supernatant using a QIAamp DNA Mini Kit (Qiagen, Hilden, Germany) according to the manufacturer’s instructions. The DNA extracts were analyzed in duplicate with the ADIAVET PARATB REAL TIME PCR kit (Adiagene, Bio-X Diagnostics S.A., Rochefort, Belgium), a detection method based on the amplification of the insertion element IS*900* of MAP, following instructions provided by the manufacturer. Data were analyzed using QuantStudio Design & Analysis Software, v1.5.1 (Life Technologies, Carlsbad, CA, USA). Cycle threshold (C_t_) values of the duplicates were determined. Values without a specific qPCR signal were assigned to 45.0. The mean C_t_ values of the duplicates were calculated and used for sample classification. Samples with mean C_t_ values ≤ 40.0 were considered positive, and values > 40.0 were considered negative.

#### 2.5.3. Dust Samples

A total of 20 mL of sterile distilled water was added to each sample and the tube vigorously vortexed. After overnight incubation at room temperature, the liquid was divided into two aliquots, and each aliquot was centrifiltrated at 3000× *g* for 5 min using an ADIAFILTER (Adiagene, Bio-X Diagnostics S.A., Rochefort, Belgium). Supernatants were discarded, and the pellets were further used, one for cultural and one for qPCR analysis as described above, with slight modifications. The pellet used for bacterial cultivation was decontaminated with only 5 mL of 0.75% hexadecylpyridinium chloride monohydrate solution (Sigma Aldrich, Taufkirchen, Germany).

#### 2.5.4. Trough Water Samples

The whole sample volume of 1 L was stepwise centrifugated in tubes of 50 mL at 4200 rpm for 20 min. The resulting pellet was suspended in 2 mL of sterile distilled water, from which 1 mL was further processed for bacterial cultivation according to the method described for the dust samples.

From samples obtained during ES 1, 2 and 3, 500 µL of the remaining 1 mL specimen were processed for qPCR analysis as described for the bedding and feed samples. From ES 4 onwards, 9 mL sterile distilled water was added to the remaining 1 mL specimen, and further DNA extraction was performed using the ADIAPURE PARATB MILK kit (Adiagene, Bio-X Diagnostics S.A., Rochefort, Belgium) according to the manufacturer’s instructions to extract MAP DNA by immunomagnetic separation. Subsequently, the extracts were analyzed in duplicate with the ADIAVET PARATB REAL TIME PCR kit (Adiagene, Bio-X Diagnostics S.A., Rochefort, Belgium) under the same conditions as described above.

### 2.6. Data and Statistical Analysis

The Kappa coefficient was calculated for dichotomized MAP detection results (positive or negative) in environmental samples using culture and qPCR to measure the agreement between both detection methods. The result was classified as described by Landis and Koch [27].

Spearman’s rank correlation coefficient was calculated to analyze the relationship between the GI (semi-quantitative culture result) and C_t_ values from qPCR sample analysis for all analyzed samples as well as each individual material if at least one positive culture result was obtained.

Characteristics listed in Table 1 were recorded for each environmental sample. The sampling sites were assigned to the following sampling locations: adult goat area (lactating goat pen, dry goat/kidding pen, buck pen), high animal traffic area (waiting area in front of the milking parlor, milking parlor, exit of the milking parlor), and youngstock area (juvenile goat pen, goat kid pen). Entrances to the barn were allocated to high animal traffic area during the grazing season and to adult goat area during the indoor season.

Logistic regression models were used to determine the likelihood of a positive environmental culture and qPCR result related to the sample characteristics. The dichotomized result of MAP detection by the respective method was set as the dependent variable. Material, location, and season as categorial, as well as the percentage of MAP shedding goats at the nearest herd examination as a numeric independent variable, were entered into the qPCR model. Because there were no cases in certain characteristic condition groups, the independent variable ‘material’ was excluded from the culture model. For the same reason, samples from the youngstock area were not included. The fit of the models was assessed using the −2 log-likelihood value.

In general, the statistical significance level was set at *p* = 0.05. Data evaluation and statistical analysis were performed using Microsoft Office Excel version 2019 (Microsoft Corporation, Redmond, WA, USA) and MedCalc version 14.8.1; (MedCalc Software Ltd., Ostend, Belgium). Figures were created using MedCalc and Microsoft Visio version 2019 (Microsoft Corporation, Redmond, WA, USA).

## 3. Results

### 3.1. Herd Examination Using Fecal Culture

Herd examination using fecal culture was conducted six times between 2018 and 2022. Only a small number of the inoculated slants showed fungal growth. However, this did not result in any sample that could not be assessed. The proportion of positive fecal samples dropped from 30.0% (January 2018) to 1.7% (May 2022) over the period of four years, but fecal shedding was present at all time points during the study period (Table 2).

### 3.2. Analysis Results of Environmental Samples

**Figure 2:**
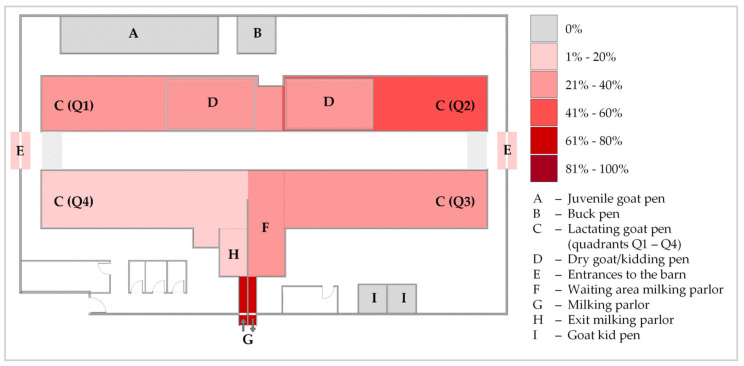
Heatmap indicating the percentage of culture-positive bedding samples for *Mycobacterium avium* subsp. *paratuberculosis* from the different sampling sites in the dairy goat barn over the study period. Dry goat/kidding pens (D) were set up temporarily within quadrants Q1 and Q2 during the kidding season in 2020 and 2021.

MAP was cultivated out of 28 (25.2%) bedding samples and one (1.8%) dust sample, whereas all water and feed samples were negative for viable MAP. This adds up to cultural MAP detection in 10.5% (29/256) of all environmental samples. Calculated GI ranged from 3.85 to 50.00 (see also Table 4). Fungal growth was partially observed in some dust samples and a few bedding samples, especially towards the end of the cultivation period. Positive culture results were obtained at 7 of 8 sampling time points. In August 2021 (ES4), cultivation of MAP was not possible from any environmental sample (Table A2). Culture-positive samples were obtained from various sites of the goat housing area but not from the youngstock area. Bacterial cultivation succeeded several times from bedding samples from the kidding pen and lactating goat pen as well as the milking area (waiting area, parlor, and exit). This area showed the most culture-positive bedding samples (47.6%), while the milking parlor was the sampling site with the highest proportion of MAP-positive samples (6/8; 75.0%). These sites, grouped together with the barn entrance during the grazing season as areas of high animal traffic, yielded 44.4% (12/27) of the culture-positive bedding samples. Bedding samples from other areas of the lactating goat pen were culturally positive in 38.1% of cases, and thus, this was the sampling site with the second most MAP detections. Kidding pens of adult goats were ranked third with 34.8% (8/23) (Figure 2). The culture-positive dust sample was collected from a kidding pen.

**Figure 3:**
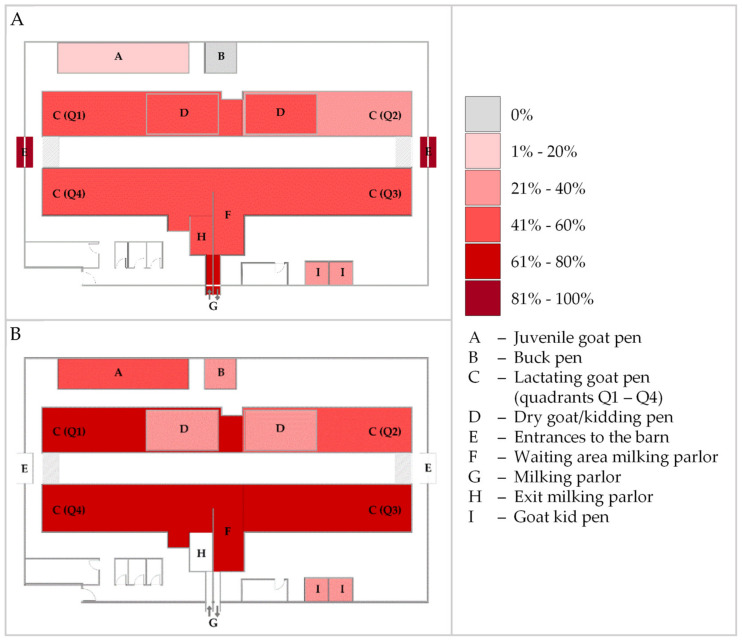
Heatmap indicating the percentage of qPCR-positive bedding (**A**) and dust (**B**) samples for *Mycobacterium avium* subsp. *paratuberculosis* from the different sampling sites in the dairy goat barn over the study period. Dry goat/kidding pens (D) were set up temporarily within quadrants Q1 and Q2 during the kidding season in 2020 and 2021. No dust samples were collected from the barn exits (E), the milking parlor (G), and its exit (H).

Positive qPCR results were obtained from 37 (64.9%) dust, 55 (49.6%) bedding, 12 (34.3%) water, and 13 (24.5%) feed samples, resulting in MAP DNA detection in 45.7% (117/256) of all environmental samples. The lowest measured C_t_ value was 27.57, obtained from a water sample. Minimum values of the other matrices were 28.58 (bedding), 31.29 (dust), and 36.11 (feed). The mean C_t_ value over all positive samples from all matrices was 35.62 (see also Table 4). During every ES, MAP DNA was detected in dust and bedding samples, whereas this was not the case for water and feed specimens (Table A2). In contrast to MAP culture, positive qPCR results were obtained from all areas of the stable, including the area of youngstock rearing and the buck pen (Figure 3).

### 3.3. Statistical Analysis

#### 3.3.1. Cohen’s Kappa

A slight agreement (ĸ = 0.20, 95% CI 0.11–0.28) [27] was observed between the MAP detection results (positive or negative) from environmental sample analysis by cultivation and qPCR (Table 3).

**Table 3:** Contingency table of *Mycobacterium avium* subsp. *paratuberculosis* detection results by culture and qPCR from 256 analyzed environmental samples used for Kappa test analysis.

qPCR Result	Culture Result		∑
	Negative	Positive	
**Negative**	135 (52.7%)	4 (1.6%)	139
**Positive**	92 (35.9%)	25 (9.8%)	117
**∑**	227	29	256

#### 3.3.2. Spearman’s Rank Correlation

**Figure 4:**
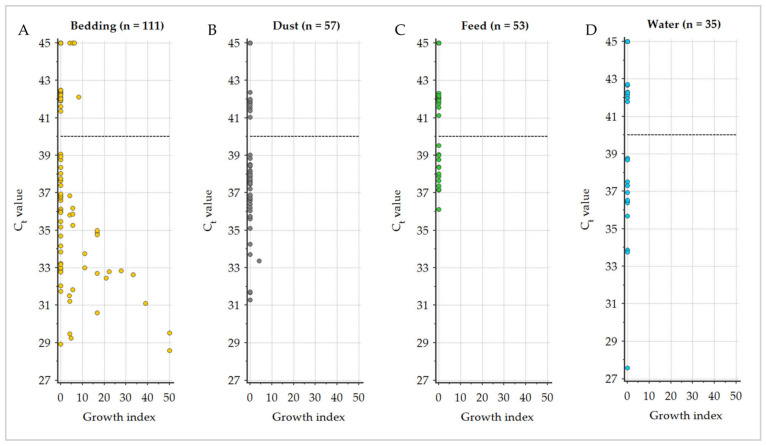
Distribution of the C_t_ values of qPCR analysis relative to the calculated growth index (semi-quantitative culture results) of *Mycobacterium avium* subsp. *paratuberculosis* in the different sampling materials (**A**): bedding, (**B**): dust, (**C**): feed, (**D**): water displayed with the qPCR classification cut-off (40.0; dashed line).

GI (semi-quantitative culture results) for cultivation on HEYM and qPCR C_t_ values of all environmental samples were significantly negatively correlated, with a corresponding Spearman’s rank correlation coefficient of ρ = −0.401 (95% CI −0.499–−0.293). Bedding samples revealed the strongest correlation (ρ = −0.529), followed by dust samples (ρ = −0.204). No correlation was calculated for feed and water samples due to complete negative culture results (Figure 4, Table 4).

**Table 4:** Spearman’s rank correlation for growth index (GI) and C_t_ values of environmental samples tested for *Mycobacterium avium* subsp. *paratuberculosis* using bacterial culture and qPCR.

Material	ρ	*p*-Value	Mean GI of Culture Positive Samples (Minimum, Maximum)	Mean C_t_ of qPCR Positive Samples (Minimum, Maximum)
All	−0.401	<0.001 *	14.64 (3.85, 50.00)	35.62 (27.57, 39.52)
Bedding	−0.529	<0.001 *	15.01 (3.85, 50.00)	34.41 (28.58, 39.08)
Dust	−0.204	0.128	4.17 (4.17, 4.17)	36.52 (31.29, 39.02)
Feed	—	—	—	38.03 (36.11, 39.52)
Water	—	—	—	35.79 (27.57, 38.78)

ρ = Spearman’s rank coefficient. *: *p* ≤ 0.05.

#### 3.3.3. Logistic Regression

The logistic regression model for positive qPCR results revealed no significant effect of the percentage of MAP-shedding goats on the likelihood of MAP DNA detection (*p* = 0.07). The odds for a positive qPCR result were higher in dust than in any other material, with an odds ratio (OR) of 2.49 compared with bedding as reference and for samples collected during the indoor season with an OR for grazing season of 0.49. Samples from feed had a lower OR (0.385) than bedding. Further, samples collected from areas of high animal traffic were associated with higher odds for MAP DNA detection (OR: 3.03) compared to those from areas where adult goats are kept, while no difference was observed between the latter and samples from youngstock areas (*p* = 0.095) (Table 5).

Another logistic regression was performed for positive culture results. The sampling location was modified by only distinguishing between high animal traffic areas and adult goat areas because MAP was not cultivated out of any sample collected from the youngstock area. The season and the percentage of MAP-shedding goats in the herd at the nearest herd examination were used in the same way as in the model described above. Of the three variables entered into the regression model, the location and season contributed significantly to the prediction of a positive culture result (*p* = 0.029; *p* = 0.034), while the percentage of MAP-shedding goats showed no significant effect. The odds for MAP cultivation were higher in samples collected from high animal traffic (OR: 3.34) compared to those from other adult goat areas. In addition, sampling during grazing season revealed a lower likelihood (OR: 0.24) for positive culture results (Table 6).

## 4. Discussion

This study was the first to evaluate the presence of MAP in various environmental materials collected from a dairy goat barn. The results allowed us to derive recommendations (1) for suitable sampling sites for the identification of infected goat herds using environmental sampling and (2) for measures to reduce MAP exposure of goat kids by classification of potential high-risk areas in the barn.

Overall, the detection of MAP in environmental samples was possible by both, qPCR and culture. This led to the conclusion that environmental samples are a suitable matrix for paratuberculosis confirmation in dairy goat herds. However, certain limitations must be considered.

MAP was only cultivated from bedding and dust, whereas samples from all materials were MAP DNA-positive. Dust samples had a significantly higher probability for qPCR-positive results than the other sampling matrices, while 96.6% of the culture-positive environmental samples were obtained from bedding. This indicates that the environmental sampling material should be selected depending on the intended analysis method.

The agreement between both detection methods was assessed as slight (κ = 0.20). This is mainly caused by 93 samples (35.9%) that were qPCR-positive but culture-negative. Fecal shedding of MAP by infected animals leads to the spread of the pathogen in the environment and, thereby, to multiple potential infection sources [5,13]. The first contact material, if present, would be bedding. Drying of the feces in combination with air circulation can lead to dissemination and sedimentary deposition. This sediment, which may or may not contain MAP, can settle on the bedding itself, on feed and water surfaces, or accumulate as dust on diverse objects in the barn [28]. The main factor for higher odds of MAP DNA detection in the dust samples is probably accumulation. Fresh forage is presented twice a day, the water in all troughs is changed daily, and new bedding material is provided when necessary. In contrast, the barn is only cleaned once a year in total, which provides a long period of time for dust to accumulate on the surfaces of barn facilities. In addition, Eisenberg et al. [14] showed that even after a cattle farm was completely destocked and cleaned with a high-pressure cleaner, MAP DNA could be found in analyzed dust samples. MAP can survive outside a suitable host for up to one year if appropriate environmental conditions are present [7]. However, in addition to viable, qPCR methods can also detect non-viable pathogens. Moreover, the existence of dormant or viable but non-cultivable states is confirmed for MAP. Dormancy describes the ability of bacteria to survive as a non-spore-forming cell without replication, mainly induced by unfavorable environmental conditions [7]. Bacteria in this reversible state may have also been detected by qPCR but caused false negative culture results.

Sample handling might also play a role in the rate of detection of culturable MAP. Freezing environmental manure samples before cultural analysis leads to lower numbers of cultivated colony-forming units per sample compared to direct sample processing [15]. In addition, decontamination during the cultivation process may adversely affect MAP viability. For samples containing small numbers of MAP which may be irregularly distributed, pellet partitioning during sample preparation can lead to false negative results of one or both analytical methods. This is demonstrated by four bedding samples from which MAP could be cultivated while being negative in qPCR. The impact of the environmental sample preparation methods used in this study on the analytical sensitivity of cultural and molecular biological MAP detection was not determined. Principle procedures were based on techniques successfully used in former studies [7,14]. A decrease of 10^2^ viable organisms was identified for soil samples spiked with feces from paratuberculosis-infected animals [13], leading to the assumption that low numbers of culturable MAP in the environmental samples may not have been detected, resulting in false negative sample classification. Taking all the mentioned factors into account, an underestimation of the true contamination of the barn environment with viable MAP is likely.

Nevertheless, a negative correlation between the C_t_ value and the GI was observed. A comparable relationship was reported for environmental manure samples from dairy cow herds in the US [15]. The Spearman’s rank correlation coefficient reported by Aly et al. [15] for frozen environmental samples (ρ = −0.611) was higher than the one calculated in the current study for all sampling materials (ρ = −0.401). However, when only bedding samples, which would be equivalent to the manure samples, were considered, the negative correlation was even stronger (ρ = −0.529), demonstrating the influence of the sample material.

Since there are no former studies concerning MAP detection in dairy goat barns, comparisons can only be made with studies conducted in cattle farms. Most of these studies analyzed mixed manure samples and, thus, areas frequented daily by large numbers of adult animals, such as alleyways and the milking parlor. In each study, these areas were identified as having the highest probability for culturable MAP [9,10,11,17,18]. Similar results were obtained in the goat housing. Here, the milking area, and especially the milking parlor, revealed the most culture-positive bedding samples. Logistic regression results underscore this finding by calculating an OR of 3.34 for the probability of bedding samples from areas of high animal traffic being positive for MAP culture compared to samples from other adult goat areas. However, viable MAP was detected in more than one-third of the bedding samples from the kidding pens of adult goats. Opposing results were reported from cattle farms where MAP was cultivated only occasionally from calving pens (14.3–17%) which were considered a sampling site with lower contamination [9,10,17,18]. The most likely explanation for this finding is the difference in the time span cows and goats normally spend in these pens. Dairy cows are usually dried off and separated from lactating cows approximately six weeks before calving. While separate dry cow and calving pens exist on most cattle farms, goats in the study herd stayed in the same pen until after kidding. In addition, in most dairy cow herds, calving pens are cleaned daily or at short time intervals, whereas in the goat kidding pen, only fresh bedding was added throughout the whole kidding season. This can lead to an accumulation of MAP even if just one shedding doe is present in the pen.

Because the probability of pathogen shedding rises with the age of the animal [5], the lack of detection of culturable MAP from samples from the kid and juvenile goat areas is plausible. Raizman et al. [18] were also unable to cultivate MAP from samples from pre-weaned calf pens, whereas 3% of samples from post-weaned calf areas were MAP-positive. This may be due to early pathogen shedding or, more likely in herds with high paratuberculosis prevalence, an introduction from the adult cow area through common devices, footwear, etc., as also mentioned by Field et al. [21], who detected MAP DNA in 21% of boot swab samples from pre-weaned calf areas. The construction modalities of the goat kid pens at the study farm, which do not require entering under normal circumstances, most likely avoid this route of pathogen entry.

However, MAP DNA was detected at every sampling site in the goat barn. This includes areas that are accessed by adult goats as well as pens where kids and juvenile goats are kept. Other studies reported comparable results for cattle husbandries [12,14]. Environmental samples from areas of high animal traffic had a higher probability of being qPCR positive (OR: 3.03) than those from other adult goat areas. No significant difference was observed between the young and adult goat area, which is in clear contrast to the culture results, where MAP was not cultivated from any sample collected in the youngstock area. Dust was the matrix with the highest proportion of positive MAP DNA results. Because the milking herd and kids are kept in one common air space, airborne distribution of MAP, viable or not, from adult to young goat areas is likely, especially because of the proximity between the milking parlor and the goat kid pens in the studied barn. Higher average C_t_ values from all materials, in particular bedding, from young compared to adult goat areas support the assumption of directed dissemination between these barn compartments. Eisenberg et al. [14] confirmed airborne distribution of MAP by pathogen detection high above the animal level where fecal contamination was unfeasible. In contrast to samples from the ground of cubicles, there was no difference concerning the likelihood of positive dust samples collected above animal level between stocked and non-stocked barn areas.

Considering the results of qPCR and cultural analysis, the milking parlor is the most recommended sampling site for both analysis methods. The farm management and husbandry conditions of the studied goat herd were very similar to those that can be found in other commercial dairy goat farms in Germany as well as other countries with a commercial dairy industry in goats. However, since samples from only one herd and barn were examined in this explorative study, the current results must be confirmed in a larger comprehensive study, including several dairy goat farms.

Two of the eight ES (ES 4 and 8) were conducted during grazing season when only the juvenile goats were in the barn throughout the whole day. The remaining dairy herd was driven inside twice a day for milking. MAP DNA was detected at every ES, while MAP could be cultivated at 7 of 8 ES. No cultivation out of any sample was possible in August 2021 (ES 4). The logistic regression model for both analysis methods identified the season (indoor vs. grazing) as a significant variable concerning the likelihood of a positive analysis result. In both cases, samples collected during grazing season had a lower probability for MAP detection (qPCR: OR = 0.49; culture: OR = 0.24). Other studies in cattle farms determined no seasonal effect [11] or reported a higher percentage of positive samples during spring and summer in comparison to autumn and winter [9]; however, some frequently positive locations were not sampled at all times of the year in the second study. The studies provide no information concerning pasture use at the farms. The data from the current study indicate that the time animals spend inside the barn is important. Prolonged stays raise the likelihood of defecation and, thereby, the risk of MAP spread by shedding animals. Furthermore, intense animal movement may contribute to dust formation and airborne distribution in the housing [12,28]. Thus, MAP detection in environmental samples will be more successful during time periods when the majority of the herd stays inside the barn continuously.

Eisenberg et al. [14] cultivated MAP from several dust and water samples from a cattle farm. These data contrast with the present study. Firstly, fecal contamination of the trough water was possible in the cattle barn, whereas this was most likely not the case in the studied goat barn. More important, in contrast to the current study, only paratuberculosis-positive cattle which had previously shed MAP were introduced into the barn. Biannual testing of the goat herd showed that during the study period, only between 9.4% and 1.7% of the animals were active shedders of MAP. As intermittent shedding is known to occur during the subclinical disease stage [3] and the herd samplings were not carried out together with the ES, these numbers only allow an approximate estimation of the precise situation at the particular ES.

Environmental manure or bedding samples are considered to be a form of pooled fecal sample due to the potential composition of feces from several animals. Thus, a dilution effect may lead to false negative results in herds with low disease prevalence [11]. Therefore, different studies tried to evaluate the correlation between MAP-positive environmental samples and disease prevalence. Donat et al. [10] estimated 87% sensitivity for the cultivation of manure samples from herds with a fecal prevalence > 2.0%. Eisenberg et al. [12] detected culturable MAP in settled-dust samples from cattle herds with low (one enzyme-linked immunosorbent-assay positive animal) but more frequently in herds with higher (two or more enzyme-linked immunosorbent assay positive animals) paratuberculosis prevalence. In the present study, logistic regression revealed no association between the number of MAP-shedding goats at the nearest herd sampling and the likelihood of MAP detection in environmental samples by culture or qPCR. A possible explanation for continuously positive qPCR results is that, despite the decreasing number of MAP shedders, the pathogen’s DNA persists in the environment for a long time and, as mentioned earlier, is not completely eliminated by normal cleaning and disinfection routines. This assumption is supported by Eisenberg et al. [12], who detected MAP DNA in more than 50% of low- and high-prevalence cattle herds. The amount of MAP shed with the feces can vary with regard to the disease stage and the individual animal [3]. It is conceivable that one high-shedding doe introduces the same number of bacteria into the environment as several light shedders, leading to consistent cultural detection of MAP. Therefore, areas that are passed by each animal are favorable for MAP detection, even in herds with only a few active shedders.

The herd examinations indicate a decreasing number of MAP-shedding goats within the herd during the course of the study. However, the data on MAP detection in environmental samples imply that a small number of active MAP shedders in the herd can sufficiently contaminate the barn environment and, thus, maintain potential infection risks.

In 2018, two years before the first ES was conducted, vaccination was included in herd management as a measure to control paratuberculosis. Despite vaccination, the barn environment was still considerably contaminated with MAP. It has to be assumed that new infections occurred in each birth cohort. The findings of this study point out that vaccination as a single measure in disease control does not lead to a complete prevention of environmental contamination with MAP and, thereby, enables the disease to maintain in the herd.

In addition to demonstrating that MAP can be found in the environment of a paratuberculosis-infected dairy goat herd, the results of the present study also allow the identification of potential risk areas for MAP transmission at the farm level. MAP was detected in dust samples in the kid-rearing area, but only by molecular biological methods. As viable pathogens are needed to cause an infection, this route of exposure is probably less critical for kids. Nevertheless, a clear structural separation of adult and young animals would be preferable in general for a further reduction of airborne MAP dissemination between these groups. However, the successful cultivation of MAP from a dust sample from the kidding area is a cause for concern. Even if one out of 57 samples is still far less than what was detected in studies in cattle farms [12,14], the sampling site is the problematic aspect. In addition to the dust sample, MAP could be cultivated from several bedding samples in this area. If kids are not immediately separated from the does after parturition, as realized using the snatching method [5,29], the likelihood of MAP intake increases over time. To minimize the environmental MAP burden of this sensitive barn area, it is recommended to set up separate kidding pens for known MAP-infected animals, to shorten the cleaning and disinfection intervals, and not to use kids from infected does for replacement.

Bedding samples from the kidding and lactating goat pens revealed a high bacterial load. This is a possible risk for contamination of the udder skin, enabling MAP intake when kids are allowed to suckle from the does. Even though a study to detect MAP on the udder skin of the goats from this herd revealed only low pathogen prevalence (10.3% qPCR-positive udder swabs) [30], the detection limits of the methods may underestimate the issue. This is another argument for the recommendation of immediate separation of the kids from the does.

The high MAP contamination of the milking parlor only plays a minor role in disease transmission since this area is only accessed by adult animals for a short time. It is assumed that a relatively high dose would be required to infect these animals [5]. In addition, environmental MAP contamination of the milk during the milking process was not detected [30]. However, the present results highlight this location as a suitable sampling site to determine the paratuberculosis-herd status using environmental samples.

Each analysis method has its specific advantages and disadvantages. When decisions about the appropriate method have to be made, the discussed key points, in combination with the objective of the investigation, should be taken into account. qPCR is an appropriate method for the identification of infected herds. Confirmation of MAP in the environment is a strong initial indicator of the presence of infected animals. MAP viability does not influence the analysis results, which increases the probability of pathogen detection. In this study, MAP DNA was detected in 117 out of 256 environmental samples, and thus, if only one sample had been taken, the MAP infection of the goat herd would have been identified in almost half of the instances. The likelihood can be increased if recommendations concerning sampling material, location, and season are considered during sample collection. Cultivation of environmental samples revealed fewer positive results (11.3%). However, MAP could be cultivated from 60.0% of bedding samples collected from high animal traffic areas during the indoor season. Therefore, this analysis method can also be suitable for paratuberculosis status determination and for confirmation of positive qPCR results. The lower limits of the within-herd prevalence of MAP-shedding animals resulting in a positive test outcome using environmental samples still have to be determined. A similarly important application of bacterial culture, however, is the identification of barn areas of high contamination with culturable MAP which serve as possible infection sites.

## 5. Conclusions

Environmental sampling can be an appropriate method for the identification of MAP-infected dairy goat herds and barn areas with a high bacterial load. To increase the probability of MAP detection, the collection of bedding or dust samples from areas with high animal traffic, especially from the milking parlor, is recommended. The applied analysis method should be chosen depending on the aim of the examination. qPCR is suitable for making a general statement about whether paratuberculosis is present in the herd or whether the direct animal environment is contaminated with MAP. Instead, cultural analysis can confirm that animals are actually shedding MAP, and identify crucial areas where the youngstock is at risk of being exposed to the pathogen and from which MAP can be spread in the barn, respectively. Detection of culturable MAP should be considered more important than solely DNA detection because of their potential role in paratuberculosis transmission. Incorporating these findings into the development of individual paratuberculosis control plans for husbandries of goats may promote efficient and effective disease control. However, to ensure the external validity of the obtained results, further research in a larger number of goat herds with different husbandry conditions and prevalence levels is needed.

## Figures and Tables

**Table 1:** Overview of recorded characteristics and conditions of environmental samples. (MAP = *Mycobacterium avium* subsp. *paratuberculosis*, HS = herd sampling).

Characteristic	Conditions
Material	Bedding
	Dust
	Feed
	Water
Location	Adult goat area
	High animal traffic area
	Youngstock area
Season	Grazing
	Indoor
Percentage of MAP shedders at nearest HS	Number culture-positive fecal samples/number analyzed fecal samples × 100

**Table 2:** Number and culture result of fecal samples collected during six examinations between 2018 and 2022 from goats older than one year.

Herd Examination	Number of Collected Samples	Positive Fecal Culture	
		*n*	%
2018 January	307	92	30.0
2020 April	286	27	9.4
2020 October	309	16	5.2
2021 May	414	13	3.1
2021 November	382	9	2.4
2022 May	407	7	1.7

**Table 5:** Outcome of the logistic regression model to estimate the relationship of the qPCR results of *Mycobacterium avium* subsp. *paratuberculosis* (MAP) to different environmental sample characteristics.

Characteristic		Positive qPCR Result		Negative qPCR Result		β ^a^	S.E. ^b^ β	*p*-Value	OR ^c^	95% CI ^d^ (OR)
		*n*	%	*n*	%					
Material	Bedding	55	49.55	56	50.45	reference	—	—	—	—
	Dust	37	64.91	20	35.09	0.913	0.363	0.012 *	2.49	1.22–5.08
	Feed	13	24.53	40	75.47	−0.965	0.399	0.016 *	0.38	0.17–0.83
	Water	12	34.29	23	65.71	−0.359	0.431	0.405	0.70	0.30–1.63
Location	Adult goat area	70	44.87	86	55.13	reference	—	—	—	—
	High animal traffic area	23	71.88	9	28.12	1.109	0.475	0.020 *	3.03	1.20–7.68
	Youngstock area	24	35.29	44	64.71	−0.563	0.338	0.095	0.57	0.29–1.10
Season	Grazing	25	40.32	37	59.68	−0.710	0.342	0.038 *	0.49	0.25–0.96
	Indoor	92	47.42	102	52.58	reference	—	—	—	—
Percentage of MAP shedders at nearest herd sampling						−0.105	0.058	0.069	0.90	0.80–1.01

^a^ β: logistic regression coefficient, ^b^ S.E.: standard error, ^c^ OR: odds ratio, ^d^ 95% CI: lower and upper limits of the 95% confidence interval. *: *p* ≤ 0.05

**Table 6:** Outcome of the logistic regression model to estimate the relationship of the culture results for *Mycobacterium avium* subsp. *paratuberculosis* (MAP) to different bedding sample characteristics.

Characteristic		Positive Culture Result		Negative Culture Result		β ^a^	S.E. ^b^ β	*p*-Value	OR ^c^	95% CI ^d^ (OR)
		*n*	%	*n*	%					
Location	Adult goat area	16	27.12	43	72.88	reference	—	—	—	—
	High animal traffic area	12	44.44	15	55.56	1.025	0.552	0.029 *	3.34	1.13–9.84
Season	Grazing	4	18.18	18	81.82	−1.443	0.681	0.034 *	0.24	0.06–0.90
	Indoor	24	37.50	40	62.50	reference	—	—	—	—
Percentage of MAP shedders at nearest herd sampling						−0.105	0.058	0.069	0.90	0.80–1.01

^a^ β: logistic regression coefficient, ^b^ S.E.: standard error, ^c^ OR: odds ratio, ^d^ 95% CI: lower and upper limits of the 95% confidence interval. *: *p* ≤ 0.05

## Data Availability

The dataset analyzed during the current study is available from the corresponding author upon reasonable request.

## Appendix A

**Table A1:** Number of environmental samples of different materials from nine sampling sites in a dairy goat barn collected at eight environmental sampling events (ES) (total *n* = 256). B = bedding (*n* = 111), D = dust (*n* = 57), F = feed (*n* = 53), W = water (*n* = 35).

Sampling Site	ES 1				ES 2				ES 3				ES 4				ES 5				ES 6				ES 7				ES 8			
	B	D	F	W	B	D	F	W	B	D	F	W	B	D	F	W	B	D	F	W	B	D	F	W	B	D	F	W	B	D	F	W
Buck pen	—	—	—	—	—	—	—	—	—	—	—	—	1	1	1	1	1	1	1	-	1	1	1	1	1	1	1	1	1	1	—	—
Dry goat/kidding pen(s)	8	2	—	—	7	2	—	—	12	3	2	—	—	—	—	—	—	—	—	—	—	—	—	—	—	—	—	—	—	—	—	—
Entrances to the barn	2	—	—	—	2	—	—	—	2	—	—	—	2	—	—	—	2	—	—	—	2	—	—	—	2	—	—	—	2	—	—	—
Goat kid pen(s)	9	7	1	7	—	1	—	—	—	1	—	—	—	—	—	—	—	—	—	—	—	—	—	—	3	3	2	1	1	1	1	1
Juvenile goat pen	—	—	—	—	3	1	1	1	—	—	—	—	1	1	1	-	1	1	1	1	1	1	1	1	1	1	1	1	1	1	—	—
Lactating goat pen	—	—	—	—	1	1	10	—	—	—	—	—	4	4	6	1	4	4	6	4	4	4	6	4	4	4	6	4	4	4	6	4
Milking rotary	1	—	—	—	1	—	—	—	1	—	—	—	1	—	—	—	1	—	—	—	1	—	—	—	1	—	—	—	1	—	—	—
Milking rotary waiting area	1	—	—	—	1	—	—	—	1	—	—	—	1	1	—	—	1	1	—	—	1	1	—	—	1	1	—	—	1	1	—	—
Milking rotary exit	—	—	—	—	—	—	—	—	—	—	—	—	1	—	—	—	1	—	—	—	1	—	—	—	1	—	—	—	1	—	—	—

**Table A2:** Number of positive samples of bacterial culture and qPCR for the detection of *Mycobacterium avium* subsp. *paratuberculosis* in environmental samples per environmental sampling event (ES) (total *n* = 256).

Analysis Result	Sampling Material	Environmental Sampling Event								∑
		ES 1	ES 2	ES 3	ES 4	ES 5	ES 6	ES 7	ES 8	
Culture positive	Bedding	5/21	3/15	5/16	0/11	5/11	6/11	2/14	2/12	28/111
	Dust	1/9	0/5	0/4	0/7	0/7	0/7	0/10	0/8	1/57
	Feed	0/1	0/11	N.s.	0/8	0/8	0/8	0/10	0/7	0/53
	Water	0/3	0/1	0/2	0/2	0/5	0/6	0/7	0/5	0/35
qPCR positive	Bedding	8/21	6/15	7/16	2/11	9/11	10/11	6/14	7/12	55/111
	Dust	7/9	2/5	1/4	6/7	3/7	5/7	7/10	6/8	37/57
	Feed	0/1	3/11	N.s.	0/8	1/8	3/8	6/10	0/7	13/53
	Water	1/7	0/1	0/2	1/2	0/5	3/6	6/7	1/5	12/35

N.s. = no sample of this material was collected.
